# Supplementary material for: Distinct sensorimotor encoding in tuft dendrites and somata associated with action, correction, and learning
Source: eLife. 2026 Sep 2;15:RP111876. doi: 10.7554/eLife.111876 (PMC13537764; doi:10.7554/eLife.111876)
Supplement: Peer review history

# Editor report: eLife Assessment

**DOI:** 10.7554/eLife.111876.3.sa0

**Authors:** Christine Grienberger

**Affiliations:** Brandeis University United States

**Keywords:** Compelling, Important

This **important** study reveals distinct representations of task-related information in the dendrites and somata of cortical neurons during sensorimotor learning and behavioral adaptation. The evidence is **compelling**, combining imaging of dendritic and somatic activity during behavior to demonstrate compartment-specific encoding of sensory cues, motor actions, and corrective signals. The work will be of broad interest to neuroscientists studying dendritic computation, motor learning, and the cellular mechanisms underlying adaptive behavior.

# Referee report: Reviewer #1 (Public review)

**DOI:** 10.7554/eLife.111876.3.sa1

[Editors' note: this version has been assessed by the Reviewing Editor without further input from the original reviewers. The authors have addressed the comments raised in the previous round of review.]

Summary:

In this manuscript, Scheib et al. identify distinct calcium dynamics in the somata and tuft dendrites of layer 5 pyramidal cells in mice performing a licking task. Animals are trained to lick water ports on the left or right following an acoustic cue, and can adjust their targeting when the ports are displaced. For tongue premotor cortical neurons projecting to the ventromedial thalamus, calcium transients in tuft dendrites are tightly locked to the direction-instructive cue, while somatic calcium signals are more broadly dispersed and more frequently synchronized with tongue motion and port contact. Finally, when the targets are shifted, tufts exhibit a sparse but large corrective signal on an improperly-targeted first lick, and the changes in population activity in the tufts and somata differ after adaptation to the new port locations.

Strengths:

In my opinion, this is a very strong manuscript which reports several novel and significant observations, contains high-quality data and (for the most part) reasonable analyses, and is clear and well-written. Most prior studies of cortical sensorimotor processing have measured the output of neurons using extracellular recording - an approach which obscures potentially important signaling differences between neuronal compartments. This study leverages cutting-edge imaging techniques in mice to document large, time-dependent differences between calcium signals at cortical somata and tuft dendrites. This phenomenon could have major implications at the cellular level for synaptic plasticity, and at the systems and behavioral levels for motor adaptation.

Weaknesses:

At a conceptual level, the authors may wish to elaborate a bit on what sensorimotor computation they think the circuit is implementing, and how their results help explain this implementation. Several possibilities are raised: tuft activation could "prime" the pyramidal cells in advance of movement initiation (line 319ff), or could track errors to engage plasticity (line 351ff) and solve the credit assignment problem (line 362ff). It might be helpful to make one of these proposals more concrete with a computational model, but this is not strictly necessary. [The authors explain that they will address this with modeling work in subsequent research.]

# Referee report: Reviewer #2 (Public review)

**DOI:** 10.7554/eLife.111876.3.sa2

Summary:

The authors set out to compare functional encoding in the tuft dendrites and somata of a specific cortical cell type during motor planning and learning.

Strengths:

The investigation of a specific projection type (L5 ET) is a strength that aids reproducibility and interpretation. The elegant approach to increasing the depth of field of dendritic imaging is another strength. The data analyses are largely clear in their methods, scope, and interpretation. The writing is extremely clear and appropriately referenced, with an excellent Introduction, in particular.

Weaknesses:

This work is largely observational, describing signals that might reflect computational transformations and/or instruct plasticity, but those possibilities have not yet been deeply investigated. The manuscript does a good job of laying out these as future directions.

# Referee report: Reviewer #3 (Public review)

**DOI:** 10.7554/eLife.111876.3.sa3

Summary:

This article by Scheib et al. investigates how layer 5 extratelencephalic (ET) neurons in the frontal cortex encode sensorimotor information during motor learning, focusing on differences between their apical tuft dendrites and somas. The authors alternated recordings among these ET neuronal compartments in the mouse anterior lateral motor cortex (ALM) during a cued directional licking task with a target port shift. They found that while tuft dendrites predominantly encode sensory cues, with a subset selectively active during corrective actions, somatic activity was more strongly associated with action timing. Additionally, learning induced divergent plasticity: tuft dendrites increased their selectivity but decreased response gain, maintaining stable net selectivity, whereas somas showed increased net selectivity early in learning. Together, these findings reveal distinct sensorimotor representations and learning-related plasticity in dendritic and somatic compartments, providing insight into how compartment-specific activity in the frontal cortex may contribute to motor skill acquisition.

Strengths:

The authors developed an innovative imaging approach and a comprehensive data analysis pipeline to address a knowledge gap in the literature. By alternating imaging of dendritic tufts and somas in the same animals, they compare compartment-specific activity during motor learning and identify distinct encoding of task variables and learning-related plasticity across these compartments. Interestingly, a subset of dendritic tufts shows activity associated with corrective actions. The findings are discussed in the context of current theories of dendritic computation, credit assignment, and motor learning, providing a useful foundation for future mechanistic studies.

Weaknesses:

No major weaknesses were identified.

# Author comment: Author response

**DOI:** 10.7554/eLife.111876.3.sa4

**Authors:** Jackson Scheib, Zachary L Newman, Jacob Gable, Deano M Farinella, Mitchell Head, Savannah Bliese, Benjamin Dougen, Harishankar Jayakumar, Sarah Young, Nicole Miller, Robert Al Khoury, Huan Kim Tran, Tien Dinh, Aaron Kerlin

**Affiliations:** University of Minnesota Minneapolis United States; University of Minnesota Minneapolis United States; University of Minnesota Minneapolis United States; University of Minnesota Minneapolis United States; University of Waikato Minneapolis United States; University of Minnesota Minneapolis United States; University of Minnesota Minneapolis United States; University of Minnesota Minneapolis United States; University of Minnesota Minneapolis United States; University of Minnesota Minneapolis United States; University of Minnesota Minneapolis United States; University of Minnesota Minneapolis United States; University of Minnesota Minneapolis United States; University of Minnesota Minneapolis United States

The following is the authors’ response to the original reviews.

**eLife Assessment**

This important study reveals distinct representations of task-related information in the dendrites and somata of cortical neurons during sensorimotor learning and behavioral adaptation. The evidence is compelling, combining simultaneous imaging of dendritic and somatic activity during behavior to demonstrate compartment-specific encoding of sensory cues, motor actions, and corrective signals. The work will be of broad interest to neuroscientists studying dendritic computation, motor learning, and the cellular mechanisms underlying adaptive behavior.

Thank you for this excellent summary. We recommend one change: removing the word “simultaneous”. It could perhaps be replaced with “concurrent” or simply omitted. Tuft dendrites and somata were imaged on alternating days, and most readers will probably interpret “simultaneous” as implying a faster, interleaved sampling rate.

**Public Reviews:**

**Reviewer #1 (Public review):**

Summary:

In this manuscript, Scheib et al. identify distinct calcium dynamics in the somata and tuft dendrites of layer 5 pyramidal cells in mice performing a licking task. Animals are trained to lick water ports on the left or right following an acoustic cue, and can adjust their targeting when the ports are displaced. For tongue premotor cortical neurons projecting to the ventromedial thalamus, calcium transients in tuft dendrites are tightly locked to the direction-instructive cue, while somatic calcium signals are more broadly dispersed and more frequently synchronized with tongue motion and port contact. Finally, when the targets are shifted, tufts exhibit a sparse but large corrective signal on an improperly-targeted first lick, and the changes in population activity in the tufts and somata differ after adaptation to the new port locations.

Strengths:

(1.1) In my opinion, this is a very strong manuscript which reports several novel and significant observations, contains high-quality data and (for the most part) reasonable analyses, and is clear and well-written. Most prior studies of cortical sensorimotor processing have measured the output of neurons using extracellular recording - an approach which obscures potentially important signaling differences between neuronal compartments. This study leverages cutting-edge imaging techniques in mice to document large, time-dependent differences between calcium signals at cortical somata and tuft dendrites. This phenomenon could have major implications at the cellular level for synaptic plasticity, and at the systems and behavioral levels for motor adaptation. As described below, I have only one major technical concern (which should be addressable with additional analysis), along with several relatively minor suggestions for improving the manuscript.

We thank the reviewer for their insightful summary of the significance of the differences that we identified in the task-related activity of tuft dendrites and somata.

Weaknesses:

(1.2) At a conceptual level, the authors may wish to elaborate a bit on what sensorimotor computation they think the circuit is implementing, and how their results help explain this implementation. Several possibilities are raised: tuft activation could "prime" the pyramidal cells in advance of movement initiation (line 319ff), or could track errors to engage plasticity (line 351ff) and solve the credit assignment problem (line 362ff). It might be helpful to make one of these proposals more concrete with a computational model, but this is not strictly necessary.

We thank the reviewer for this feedback. We absolutely agree that detailed computational models of each proposed computation will be very valuable and constitute an important follow-up to this work. We hope to collaborate with theorists to take that next step. Each possible computation noted by the reviewer reflects distinct differences that we observed in the task-related activity of tuft dendrites and somata. They are not mutually exclusive hypotheses to explain the same phenomenon. As such, we think they are best addressed independently in future modeling work. By making the data and a concise description of the main findings available immediately, we hope to allow computational experts in each of these areas to take advantage of the results of this study without delay.

(1.3) My only major technical concern relates to the analyses in Figures 4F-H, 5G-I, and 6H-K (c.f. equations 2-5). Typically, one identifies population-level factors by projecting neural activity onto fixed dimensions of interest; this makes it possible to see how activity evolves over time along interpretable coordinates. Here, however, the coding directions are redefined at each time point, so the "choice" activity at time t is actually a different signal from the "choice" activity at t+1. This procedure is a bit like comparing the activity of one neuron at one time point with the activity of a different neuron at a later time point. It also makes the physiological interpretation more complicated: if the dimensions are fixed, one can see how a downstream neuron could "read out" the signal by computing a weighted sum of the activity of upstream neurons, but it is harder to see how this could happen if the weights are always rotating.

We thank the reviewer for raising this point. We agree that our use of projections along coding directions (CDs) defined at each time point is a less conventional use of coding directions, although nearly identical calculations have been previously used to assess population-level selectivity and code stability in this task (Chen et al., 2017; Yang et al., 2022). As noted in the article, given low numbers of error trials and high trial-to-trial variability, we found that estimating the selectivity of individual ROIs for these task-dimensions was not robust and was subject to overfitting. Cross-validated projections at each timepoint provided a far more robust measure of population selectivity. Furthermore, we were able to orthogonalize stimulus, choice and outcome CDs to better identify distinct encoding of each task-variable. Finally, because the primary goal of the study was to identify any differences between tuft dendrite and somatic encoding, we think that calculating the population selectivity at each timepoint gives readers a less biased view of the selectivity of the two compartments, whereas calculating a CD over a single arbitrary time window could conflate differences in dynamics with differences in selectivity.

We agree that calculating the CD at each timepoint makes it hard to see where the code is stable and where it is rotating, and thus how a downstream neuron might “read out” the signal. To provide this information, we have added new panels to the supplement showing the correlation of CDs across time (Figure 4 - figure supplement 1B,D). We also now provide this information for CR-CA in Figure 5—figure supplement 2A (the plots previously presented in 2A were the correlations of CR with CA, rather than CR-CA; an error that has been fixed). The following changes were also made to the Results section to clarify this issue:

“From the linear model, we calculated coding directions (CDs) at each timepoint that maximally separated Stimulus, Choice, and Outcome activity (Figure 4F; Figure 4—figure supplement 1A) and estimated the direction and selectivity along each dimension across time (Figure 4—figure supplement 1B-E; see Methods). Allowing CDs to rotate in time (see Figure 4—figure supplement 1B,D), although unconventional, ensured that comparisons of population selectivity across the two compartments were not biased by the selection of an arbitrary CD time window.”

We also identified a mistake in the description of CD orthogonalization in the Methods, which has been corrected as follows:

“For each timepoint, each selectivity CD was then orthogonalized with respect to the other two selectivity CDs by a QR decomposition in which that selectivity CD was last in the order.”

(1.4) A few comments on the behavioral task and results. After the port shift, the error rate is quite high, and doesn't diminish much between the early and late epochs (approximately 42% and 38% error rate, respectively; Figure 1I). That is, mice do not seem to fully master the task. Clearly, animals do alter their aim, but even this does not seem to change much between early and late periods (Figure 1J). I recommend that the authors show the behavioral data at a finer level of granularity (e.g., by plotting the change in exit trajectory on all individual trials across sessions, with a loess fit) to allow an assessment of the adaptation rate and when adaptation saturates. It would also be more conventional to refer to the behavioral changes as "motor adaptation," instead of "skill learning." (The latter would be appropriate if the port offset were randomized across trials, and animals received two separate cues for direction and offset, but I suspect this task would be too difficult for mice to learn.)

We agree with the reviewer that by the end of the late period, performance on the right side (Figure 1I) has still not returned to pre-shift levels. This may reflect mice not fully mastering the task, as the reviewer suggests, or it may reflect that after the shift, the right port is substantially more difficult to reach than the left port. Unfortunately, because of high animal-to-animal and lick-to-lick variability, plotting the post-shift lick angle at a finer level of granularity is not statistically informative.

With regard to the nature of the learning in our task, we selected “skill learning” as the best description of the motor learning task based on distinctions between adaptation and the learning of motor skills by Krakauer et al., 2019 and Heald et al., 2021. Conceptually, the difference is whether an existing motor controller memory is simply updated with new parameters, or whether the motor context has changed sufficiently that a distinct motor controller memory (which can still use parts of previous memories) is formed. In our task, after the port shift the left port forms an obstacle to reaching the right port. This obstacle was simply not present before the shift. Before the shift, ports were approximately equidistant from the mouth and easily avoided given the port separation and tongue width. Thus, avoiding an obstacle would presumably not be part of the initial motor controller memory and a distinct memory would need to be constructed.

We agree with the reviewer, however, that given that we do not have fine-timescale dynamics of behavioral changes in response to the shift, and did not conduct other experiments (such as returning the ports to their original location) that would typically be conducted to identify “adaptation-like” or “skill learning-like” dynamics, we cannot empirically distinguish between the two. We now clarify in “Study limitations” that we call the studied behavior “skill learning” based on the nature of the task, but that our behavioral analysis cannot distinguish between adaptation and skill learning:

“We refer to the behavioral paradigm as motor “skill learning” strictly based on the nature of the task. After the shift, mice must avoid a new obstacle close to the mouth (i.e., the left port), which we assume requires the formation of a distinct motor controller memory and therefore would be considered skill learning (Krakauer et al., 2019). However, we did not confirm that the mice exhibited specific behavioral characteristics of skill learning and it is possible that other kinds of motor learning (e.g., motor adaptation) were dominant.”

(1.5) This is perhaps a semantic point, but it might not be entirely accurate to refer to the activity evoked by the directional cue as "sensory." Typically, a "sensory" response should encode some feature of a stimulus - in this case, the frequency of a tone. Here, it seems likely that the cue-aligned activity reflects the instructed lick direction, rather than the auditory information per se. (Presumably, these premotor neurons do not have well-behaved auditory tuning curves.) By comparison, in macaques performing center-out reach tasks, activity in dorsal premotor cortex rapidly ramps up following a visual cue instructing the direction of an upcoming reach, but one usually wouldn't refer to this activity as "visual" or "sensory" (though this is sometimes done). I suggest the authors either use "Instruction" or similar (e.g., in Figure 4F), or clarify in the text whether they think the activity is a genuine auditory response or something else.

We understand how this could cause confusion. “Sensory” was meant to denote the nature of the differences in external events between the trial types used to calculate selectivity, not to imply that the activity was necessarily selective for detailed features of the cues outside the context of the task. Previous work in ALM cortex has labeled this selectivity direction as “stimulus” (Yang et al., 2022; Chen et al., 2024) to better emphasize that it is simply defined by the external cue. Where appropriate, we have revised the article to use “stimulus” or “instructional cues” in place of “sensory” for clarity and to better conform with convention.

**Reviewer #2 (Public review):**

Summary:

The authors set out to compare functional encoding in the tuft dendrites and somata of a specific cortical cell type during motor planning and learning.

Strengths:

(2.1) The investigation of a specific projection type (L5 ET) is a strength that aids reproducibility and interpretation. The elegant approach to increasing the depth of field of dendritic imaging is another strength. The data analyses are largely clear in their methods, scope, and interpretation. The writing is extremely clear and appropriately referenced, with an excellent Introduction, in particular.

We thank the reviewer for their appreciation of the study design, imaging methods, and scholarship of the article.

Weaknesses:

(2.2) It is not obvious whether the selected labeling strategy avoids labeling Layer 6 CT neurons, which would contaminate dendritic recordings. The images provided suggest enrichment in L5, but a discussion of this important potential caveat is warranted, especially since within-cell comparisons of apical dendrites to somata were not performed.

We thank the reviewer for emphasizing the need to discuss this potential issue. For the following reasons, it is likely that the vast majority of dendrites we imaged in layer 1 originated from layer 5 ET neurons. First, as the reviewer notes, the provided images suggest enrichment in layer 5. This enrichment likely reflects the fact that most L6 CT neurons in motor and premotor cortex send denser projections to other thalamic nuclei than to VM thalamus (Winnebust et al., 2019, Cell), where we targeted our retrograde-Cre injections. Second, L6 CT neurons are predominantly untufted (Ledergerber and Larkum, 2010, J. Neurosci.), including in motor and premotor cortex (Peng et al., 2021, Nature; Ichikawa, 2025, Front. Neuroanat.). A recently identified subclass of L6 CT neurons in secondary motor cortex has dense projections to VM thalamus, but this class also appears to extend minimal dendrites into L1 (Li et al., 2024, bioRxiv). Nonetheless, we did not label post-hoc tissue collected from imaged mice with markers of precise laminar boundaries, and thus cannot definitively rule out the possibility that dendrites from a subclass of L6 CT neurons with tuft dendrites were also imaged. We have added the following paragraph to the “Study limitations” section to make readers aware of these issues:

“L5 ET neurons in premotor cortex elaborate extensive tuft dendrites in L1, whereas Layer 6 (L6) corticothalamic (CT) neurons are predominantly untufted (Jiang et al., 2020; Peng et al., 2021). Thus, although we cannot rule out the possibility that dendrites from a subclass of L6 CT neurons were also sampled, it is likely that the vast majority of dendrites we recorded in L1 originated from L5 ET neurons.”

(2.3) The application of DeepInterpolation to dendritic data appears to be novel, and little detail or vetting is provided. The reader is left guessing: Was the model retrained or fine-tuned on dendritic data? How does the denoising affect the resulting segmentation and activity traces? Is denoising necessary for this workflow?

We thank the reviewer for requesting this useful additional information.

In all cases, the model was retrained for each dendritic or somatic imaging session. Denoising improved segmentation consistency, as measured by comparing segmentations of individual sessions from the same animal. This is now specified in the Methods as follows:

“The DeepInterpolation model was trained on each imaging session prior to denoising of that session. Denoising prior to NMF-based segmentation resulted in more robust and consistent dendrite segmentation than NMF-based segmentation without prior denoising (0.79 +/- 0.01 ⍴ vs. 0.46 +/- 0.01 ⍴; mean of the max Spearman correlation of components across sessions; random subsample of N = 3 mice, 15 sessions, 400 components).”

With regard to how denoising impacts activity traces, examples were shown in Figure 2I, K. To provide more quantitative information to the reader, we calculated estimates of the power and reliability of the spectral content of dendrite activity traces extracted with or without denoising. These data are now shown in the new panel, Figure 2 - figure supplement 2G. The power spectral density of the denoised activity and the estimated reliable power spectral density of the raw traces match up to approximately 2.6 Hz (Figure 2 - figure supplement 2G), which is not far from the bandwidth of GCaMP8m, given its estimated combined rise and decay (Figure 2 - figure supplement 3B, C). Some frequencies beyond this point have been suppressed beyond what would be expected due to photon shot noise (as estimated by the replicate coherence-weighted PSD, or “recoverable” PSD). Further characterization of the precise nature of the suppressed high-frequency information – which could be suppressed artifacts (e.g., fast brain motion) or lost signal detail (i.e., GCaMP8m rise kinetics) – is beyond the scope of this paper.

Details of the PSD calculations have been added to the Methods, and the following statement has been added to the Results: “Power spectral density of the denoised traces and the coherence-weighted power spectral density of the raw traces match up to approximately 2.6 Hz (Figure 2 - figure supplement 2G; Methods), which is not far from the bandwidth of GCaMP8m, given its estimated combined rise and decay (Figure 2 - figure supplement 3B, C).”

(2.4) The activity patterns of the recorded cells appear to lack the characteristic ramping during the delay epoch previously reported in both calcium imaging and electrophysiology studies. Given that a major contribution to the significance of the work is to constrain models of ALM function, a discussion of how the data aligns with previous measurements in the same circuit would improve the work.

Preparatory selectivity and ramping activity can be seen in Figure 3H, Figure 6I, and Figure 5 – figure supplement 1B. We note that in ALM cortex, the ramping mode explains a minority of the total variance (~17%, Yang et al., 2022), but it can appear particularly prominent in projections along certain fixed CDs.

(2.5) It would be very informative to compare differences in signals between dendrites and somata of the same cells. Consistently tracing dendrites to their respective somata would assuage worries of potential contamination from dendrites of deeper cells and enable more direct comparisons of signal transformations between dendrites and somata. It would be good to understand the relationship between dendritic calcium signals and backpropagating action potentials in this task. The authors detect less frequent calcium events in tufts versus somata; is this due to selective backpropagation of action potentials? The dynamics of this process were recently investigated by Adam Cohen's group *in vivo* and *in vitro*, and measurements in the present settings could be compared to such work.

We agree with the reviewer that being able to compare differences in signals between the dendrites and somata of the same cells would be very valuable. However, reliable tracing of tuft dendrites to somata from *in vivo* 2P anatomical imaging requires extremely sparse labeling, such that very few neurons are recorded per animal (Kerlin et al., 2019, eLife; Otor et al., 2022, Science). As stated in the “Study limitations” section of the Discussion, we suspected (correctly) that some task-related selectivity (i.e., selectivity for corrective action) would be sparsely represented in the dendrites, and thus adopted a labeling and image processing strategy that allowed us to record from many dendrites per animal. This strategy necessarily comes at the expense of generating a labeling density that precludes reliable tracing of tuft dendrites to their respective somata based on 2P morphology alone. As discussed in our response to reviewer comment 2.2 and a new paragraph of “Study limitations,” substantial contamination of the dendrite recordings by dendrites of L6 CT neurons is highly unlikely. Future studies could use simultaneous functional imaging across large volumes combined with activity-based segmentation or post-hoc high-resolution imaging of tissue sections registered to *in vivo* 2P imaging to accomplish both high-throughput dendritic imaging and reliable tracing.

We thank the reviewer for pointing out that we could discuss selective backpropagation as a potential mechanism more explicitly. Our results are consistent with previous studies of L5 tufts *in vivo* (Francioni et al., 2019, eLife), including in ALM cortex (Maristany de las Casas et al., 2026, Science), that reported that rates of multi-branch calcium transients in the tuft dendrites of L5 neurons are lower than somatic spike rates. As discussed in “Study limitations,” there is not a clear approach in our data to determine the precise nature of the events underlying the calcium transients we measured in the tuft dendrites. Selective backpropagation of action potentials is certainly one possibility and we agree that recent research from Dr. Adam Cohen’s group should be discussed. We have added the following to the Discussion:

“Based on previous calcium imaging of L5 tufts in ALM cortex of mice engaged in similar tasks (Kerlin et al., 2019; Maristany De Las Casas et al., 2026), we suspect that most of the activity we measured was coincident with global tuft or hemi-tree events, as well as somatic spiking. Recent *in vivo* voltage imaging in the hippocampus has also indicated that most spikes in distal dendrites start as bAPs that have been selectively amplified (Wu et al., 2026; Lee et al., 2026).”

(2.6) The Coding Direction analyses presented in this work, while consistent with previous literature on population codes in ALM, are at odds with the nature of the measurements here. The changes in representation that occur between the dendrites and soma of an individual cell are probably best thought of in terms of the dynamics of signals themselves within individual neurons, rather than in the information encoded across a population.

We thank the reviewer for giving us the opportunity to clarify this issue. As noted in the article, given low numbers of error trials and high trial-to-trial variability, we found that estimating the selectivity of individual ROIs for these task dimensions was not robust and was subject to overfitting. Cross-validated projections at each timepoint provided a far more robust measure of population selectivity. Furthermore, we were able to orthogonalize stimulus, choice and outcome CDs to better identify distinct encoding of each task variable in the population activity. Thus, the analyses are not at odds with the nature of the measurements in the study.

Nevertheless, it is true that by recalculating the CD at each timepoint, our selectivity projections do not provide the same information as conventional projections along a fixed CD, which can indicate where the selectivity code is stable and where it is changing. To provide this information we have added new panels to the supplement showing the correlation of selectivity CDs across time (Figure 4 - figure supplement 1B, D).

(2.7) This work is largely observational, describing signals that might reflect computational transformations and/or instruct plasticity, but those possibilities have not yet been deeply investigated. The manuscript does a good job of laying out these as future directions.

We agree with the reviewer. As noted by the reviewer in comment (2.1), we combined a number of approaches in an innovative manner to explore how tuft dendrite activity differs from somatic activity at the population level during motor learning. These measurements provide the necessary foundation for future mechanistic studies and we think it is appropriate to share them at this stage of investigation and in the format of this article.

**Reviewer #3 (Public review):**

Summary:

This article by Scheib et al. investigates how layer 5 extratelencephalic (ET) neurons in the frontal cortex encode sensorimotor information during motor learning, focusing on differences between their apical tuft dendrites and somas. The authors alternated recordings among these ET neuronal compartments in the mouse anterior lateral motor cortex (ALM) during a cued directional licking task with a target port shift. They found that while tuft dendrites predominantly encode sensory cues, with a subset selectively active during corrective actions, somatic activity was more strongly associated with action timing. Additionally, learning induced divergent plasticity: tuft dendrites increased their selectivity but decreased response gain, maintaining stable net selectivity, whereas somas showed increased net selectivity early in learning. Together, these findings reveal distinct sensorimotor representations and learning-related plasticity in dendritic and somatic compartments, providing insight into how compartment-specific activity in the frontal cortex may contribute to motor skill acquisition.

Strengths:

The authors developed an innovative imaging approach and a comprehensive data analysis pipeline to address a knowledge gap in the literature. By alternating imaging of dendritic tufts and somas in the same animals, they compare compartment-specific activity during motor learning and identify distinct encoding of task variables and learning-related plasticity across these compartments. Interestingly, a subset of dendritic tufts shows activity associated with corrective actions. The findings are discussed in the context of current theories of dendritic computation, credit assignment, and motor learning, providing a useful foundation for future mechanistic studies.

We thank the reviewer for highlighting interesting findings in the paper and their assessment that it provides a “useful foundation for future mechanistic studies”.

Weaknesses:

No major weaknesses were identified.

**Recommendations for the authors:**

**Reviewer #1 (Recommendations for the authors):**

A very minor suggestion: it would be useful to mention the model organism in the abstract or title.

(1.6) Thank you for catching this. We have added the model organism to the abstract as follows:

“Using longitudinal two-photon calcium imaging, we investigated sensorimotor encoding in the apical tuft dendrites and somata of L5 extratelencephalic (ET) neurons in the frontal cortex of mice during learning of a discrete change to a cued dexterous action.”

**Reviewer #3 (Recommendations for the authors):**

Major:

(3.1) Lines 197-199: It is unclear why the authors conclude that somas have stronger representations of choice and task outcome. In Figure 4G, there is no significant difference between dendrites and somas for Choice or Outcome coding selectivity. The differences in the Sensory/Choice and Sensory/Outcome ratios shown in Figure 4H,I are likely explained by stronger Sensory selectivity in dendrites (Fig 4g), rather than by stronger Choice or Outcome encoding in somas.

We agree with the reviewer’s interpretation of the data. The statement at 197 - 199 was meant to reflect relative selectivity, but it was imprecise. We have replaced that sentence with the following, more precise sentence:

“Somatic activity also encoded these features, but the representation of the stimulus was weaker – and the representation of action timing was stronger – than in the tuft dendrites.”

(3.2) Figure 4B: The authors realign FL-associated IRFs to GO-cue timing using the mean FL latency for each trial type and animal. Because FL timing is jittered across trials and may differ between CL and CR trials, this could smear the realigned traces and complicate the interpretation of contact-associated activity. The authors should consider using trial-by-trial FL timing for realignment or quantify the impact of FL-timing variability on the resulting traces.

We aligned average GO- and contact-IRFs in Figure 4B so that comparisons of their magnitudes could be drawn from the same time window.

With regard to jitter across trials, we think the reviewer may have misinterpreted how the mean IRFs in Figure 4B are calculated. The contact-IRF, by its nature, is calculated once per animal and trial type with respect to FL timing and shifted once based on mean FL latency. There is no smearing due to trial-to-trial FL timing.

With regard to systematic differences in FL timing across animals and CR vs. CL, the reviewer is correct that this could – in theory – smear the realigned mean contact-IRF shown in Figure 4B. However, differences in mean FL latency across animals and trial-types are small compared with the long-timescale contact-IRFs. Thus, the non-realigned (i.e., always FL-aligned) mean contact-IRF looks nearly identical to Figure 4B just globally offset in time, as shown in Author response image 1:

**Author response image 1.** 

Since this is nearly identical to data already presented in Figure 4B, we do not think it is necessary to include it in the revised article. However, we have added the following to the Methods:

“Population averages of contact-IRFs that were not shifted prior to averaging were nearly identical (excluding the overall temporal shift; data not shown), indicating that pooling of mean IRFs across animals and trial types produces minimal smearing of the final population IRF.”

(3.3) Figure 5A: Are CA trials specific to motor learning, or do they reflect a corrective lick toward the alternative port after an unrewarded lick? An analysis of the second lick on left-error trials or pre-shift right-error trials could help distinguish whether correction licking reflects a general decision change after failed reward, or a motor-command correction specific to post-shift motor learning. The authors should also report the prevalence of CA versus AP trials and clarify whether these trial types are behaviorally distinct.

We thank the reviewer for highlighting the need to emphasize that CA trials reflect a distinct behavior related to reaching the displaced port.

By definition, CA trials started as Motor Error trials and thus reflected a corrective lick toward the same port after an unrewarded lick. Almost all first contact licks on Motor Error trials were well outside the distribution of correct left licks both pre- and post-shift (Figure 1 - figure supplement 1B,D), consistent with the interpretation of this first lick as directed toward the right port. Thus, we see no evidence suggesting that CA trials involve a decision change. CA trials are exceedingly rare pre-shift, because Motor Error trials are rare pre-shift (Figure 1I, only ~5% of all right trials).

With regard to other error types before the shift, most expert-trained mice did not immediately sample the other port with a second lick after an unrewarded lick. They usually either stopped licking immediately or licked the unrewarded port multiple times before switching ports. When port switches occurred pre-shift, timing was highly variable across mice and trials. Even on rewarded trials, some mice would “check” the unrewarded port after consuming the reward, as can be seen in Figure 3I, J. All of these behaviors are clearly distinct from the stereotyped second lick that occurred on CA trials after the shift. We agree that the prevalence of CA and AP trials, as well as the prevalence of immediate port alternation, should be reported, and we have added that information to the article as follows:

“On Correction Attempted (CA) trials, the first lick made contact with the incorrect port, and the mouse chose to direct a second lick toward the correct port (Figure 5A; prevalence: 54% of motor error trials). We interpreted these licks as a corrective action, because the tongue exit angle shifted further toward the correct target (Figure 5B). Abandoned Port (AP) trials were the same as CA trials, except the mouse either did not make a second attempt or the second lick was directed toward the incorrect port (Figure 5A; prevalence: 46% of motor error trials).”

(3.4) The classification of pre-shift errors into motor and decision errors is not clear. If error-trial exit angles follow a unimodal distribution (Figure 1- Figure Supplement 1C), then the distinction between motor and decision errors may not be behaviorally well separated. The authors should explain how these categories are validated and whether conclusions depending on this classification are robust to alternative definitions.

We do not conclude that motor errors and decision errors are distinguishable pre-shift. Pre-shift licks were classified into motor error and decision error categories only to demonstrate that the boundary we established for classifying post-shift licks classifies extremely few (~5%, Figure 1I) pre-shift licks as motor errors. No conclusions were drawn from comparisons between pre-shift licks classified as decision errors and those classified as motor errors. The categorization is defined by the distribution of exit angles pre-shift and validated by the bimodal distribution of exit angles on error trials post-shift. To improve clarity regarding our classification of pre-shift errors, we have added the following to the Results:

“Exit angles after the shift exhibited a bimodal distribution across error trials (Figure 1G,H; Figure 1—figure supplement 1C,D), supporting this distinction in error type. The frequency of licks classified as motor errors on right-cued trials increased significantly after the shift (median pre-shift 0.06, median post-shift 0.42, *p* < 0.001; Figure 1I; Figure 1—figure supplement 1C,D), reflecting the new challenge of avoiding the left lickport. In contrast to after the shift, exit angles on error trials before the shift were unimodal (Figure 1—figure supplement 1C). These errors were classified based on the fixed CB in order to demonstrate that very few pre-shift licks qualify as motor errors (Figure 1I), and not to suggest that tongue trajectories before the shift are behaviorally well-separated.”

(3.5) Figure 1- Figure Supplement 1D, post-shift decision errors: Are these truly decision errors? The lick angles appear similar to those observed before the shift, suggesting that these trials may reflect execution of a "default" or "uncertain" lick trajectory rather than an incorrect choice under the new contingency.

The post-shift exit angles on right-cued decision error trials (Figure1 - figure supplement 1D, grey) are similar to the lick angles on correct left-cued trials pre-shift (Figure 1 - figure supplement 1A, red) and clearly different from the correct right-cued trials pre-shift (Figure 1 figure supplement 1A, blue). Thus, to the extent that the animal’s intention can be measured from lick trajectory, it was targeting the incorrect (left) port. It is also true that it may still target the previous location of the left port (a “default” left trajectory), but because the decision error makes precise targeting irrelevant to the task outcome (it is easy to reach the left port after the shift), we do not designate it as a joint decision error and motor error. As to whether the deliberative process leading to this action is somehow cognitively distinct from other behaviors typically labeled as decision errors or incorrect choices, we cannot say.

Minor:

(3.6) Vocabulary consistency: soma vs somata.

When data are shown for, or derived from, multiple somata, we use “somata”. When data are shown for an individual soma (such as in a panel with data from a single example soma), we use “soma.” We could not find any use of “somas,” which would indeed be inconsistent.

(3.7) Figure 1C: I am not sure why the lick trajectories do not depict the tongue exiting the mouse. What time window is shown? Why does it look like the trajectories are shifted to the left?

We thank the reviewer for identifying this issue. The definition of the location labeled “mouth” was accidentally omitted. The lick trajectories in Figure 1C do depict the tongue tip once it became visible to the cameras. Jaw opening and shifting partly determined the location where the tongue became visible in the videography. These movements varied from mouse to mouse and trial to trial, so exit angle was measured from the approximate midpoint between the temporomandibular joints, which is the grey point in 1C. We have fixed the captions and Methods to precisely define this location. With regard to the appearance of a slight leftward shift in the trajectories, this reflects how the tongue exits the mouth and how the tongue tip curves downward as the tongue approaches the port.

(3.8) Figure 1- Figure supplement 1: it could ease the comparisons to report population statistics, such as median, from panel A to panel B and D, population statistics from B to D.

Thank you. We have added these statistics to the Figure 1 - figure supplement 1 caption.

(3.9) Choice boundary (CB) should be defined in line 100, not 110.

Thank you. We have fixed this.

(3.10) Line 109: claim not supported by referenced figure (Figure 1 - Figure Supplement 1). Lick angle histogram to the right port, pre-shift does not overlap substantially with lick angle to the left port, post-shift.

We thank the reviewer for the opportunity to clarify this. We agree that Figure 1 - figure supplement 1 is not sufficient to support the claim. First, we want to make clear that Figure 1 - figure supplement 1 does not contradict the claim. The new location of the left port can obstruct the tongue during right-cued licks, regardless of the distributions of left licks pre- or post-shift. Second, to confirm that the new location of the left port would obstruct a substantial fraction of pre-shift right-cued lick trajectories, we measured the minimum distance between tongue trajectories and the post-shift location of the left port. Of pre-shift right-cued exit trajectories, 30 +/- 5% came within 1.25 mm – half of the combined tongue width (1.5 mm) and port width (1 mm) – of the port center.

To make this claim more precise, we have changed the statement as follows:

“Thus, on right-cued trials, mice continuing to follow the pre-shift motor plan would be biased to more frequently contact the new left port location (Figure 1E,F; 30 +/- 5% of pre-shift trajectories came within a tongue-width of the new location) and receive punishment (i.e., timeout).

(3.11) Line 113: claim not supported by referenced figure. Figure 1G does not display error trials.

We have changed the line to refer to “both correct and error trials”, such that reference to Figure 1G is also appropriate.

(3.12) Figure 2 - Figure Supplementary 3 & method: how is noise estimated?

Thank you. The following has been added to the Methods:

“For Figure 2 - figure supplement 3, noise was estimated as the square-root of the geometric mean of the Welch power spectrum in a high-frequency band (0.25–0.5 times the frame rate; Giovannucci et al., 2019).”

(3.13) Figure 2D: Was imaging during the shift epoch always performed in dendrites? If so, could the imaging schedule bias comparisons between dendritic and somatic activity during learning, especially given that mice show behavioral learning between early and late post-shift sessions (Figure 1J)?

No, imaging during the shift was not always performed in the dendrites. The following has been added to the Methods to make clear that the post-shift data reflect dendritic and somatic imaging conducted on the day of the shift with roughly similar frequency:

“For Figure 5 and Figure 6, which make comparisons between dendritic and somatic activity during the post-shift period, 67% of animals providing somatic data (4 of 6 mice) underwent somatic imaging on the day of the shift and 80% of mice providing dendritic data (8 of 10 mice) underwent dendritic imaging on the day of the shift.”

(3.14) Lines 163-164, "we observed that the onset of tuft activity was consistently time-locked to the GO cue (vertical green line; Figure 3B). This was in contrast to somatic activity, which had more variable timing (Figure 3E)." The authors cite panels B and E in support of this point, but these appear to be example ROIs. It would be helpful to clarify how representative these examples are, since the corresponding population summaries in panels G and H do not make the effect immediately apparent.

These examples are representative, as supported by the population summary of activity time-locked to the GO-cue versus port contact in Figure 4B.

(3.15) Figure 4B: It could be useful to add the lick traces here as well. To allow the reader to have an idea of contact timing with respect to the Go cue and compare the sustain response with the licking pattern.

We understand how this could be helpful. However, since these exact traces are already present in Figure 3I,J, we think that adding them to Figure 4 is unnecessary and would add complexity to an already very busy figure.

(3.16) Figure 5E: Why are the imaging sessions labeled 0 and +1 rather than 0 and +2? Are the dendritic and somatic imaging not alternated?

Yes, imaging was not alternated for 3 of the 22 mice. We have clarified this in the Methods, as follows:

“Somatic and dendritic imaging sessions alternated every other day (19 of 22 mice), except for 3 mice in which only one compartment was imaged daily (dendrite-only: 2 mice, soma-only: 1 mouse). The exceptions were due to brain curvature or the angle of the coverslip with respect to the brain, such that only one compartment could be imaged and the other compartment was underneath skull regrowth or dural thickening that made high-quality imaging impossible.”

(3.17) Figure 6C, legend: I suppose the authors meant "remapping", not "Post-shit SI distribution" for the description of the right column.

Thank you. We have fixed this label.
